# The influence of *Streptococcus pneumoniae* nasopharyngeal colonization on the clinical outcome of the respiratory tract infections in preschool children

**DOI:** 10.1186/s12879-015-1149-8

**Published:** 2015-09-30

**Authors:** Sigita Petraitiene, Tomas Alasevicius, Indre Staceviciene, Daiva Vaiciuniene, Tomas Kacergius, Vytautas Usonis

**Affiliations:** Clinic of Children’s Diseases, Faculty of Medicine, Vilnius University, Vilnius, Lithuania; Children’s Hospital, Affiliate of Vilnius University Hospital Santariskiu Klinikos, Vilnius, Lithuania; Department of Physiology, Biochemistry, Microbiology and Laboratory Medicine, Faculty of Medicine, Vilnius University, Vilnius, Lithuania

**Keywords:** *Streptococcus pneumoniae*, Nasopharyngeal colonization, Preschool children, Respiratory tract infection

## Abstract

**Background:**

*Streptococcus pneumoniae* (SPn) is an important pathogen causing a variety of clinical manifestations. The effects of SPn nasopharyngeal colonization on respiratory tract infections are poorly studied. We evaluated the association of SPn colonization with features of respiratory tract infections.

**Methods:**

Children under the age of 6 years who visited a primary care physician because of respiratory tract infections were enrolled in the study. History was taken, children were clinically assessed by the physician, and nasopharyngeal swabs were obtained and cultured for SPn. Positive samples were serotyped. Associations of SPn colonization with clinical signs and symptoms, recovery duration, absence from day care centre, frequencies of specific diagnoses, and treatment with antimicrobials were evaluated.

**Results:**

In total 900 children were enrolled. The prevalence of SPn colonization was 40.8 % (*n* = 367). There were minor differences between male and female subjects (199 of 492, 40.4 % vs 168 of 408, 41.2 %, *p* = 0.825). Children with and without siblings had similar colonization rates (145 of 334, 43.4 % vs 219 of 562, 39.0 %, *p* = 0.187). Clinical signs and symptoms were not associated with SPn colonization. Children colonized with SPn had longer recovery duration compared to non-colonized children (114 of 367, 31.1 % vs 98 of 533, 18.4 %, *p* < 0.001) and were longer absent from day care (270 of 608, 44.4 % vs 94 of 284, 33.1 %, *p* = 0.001). Pneumonia, sinusitis, and acute otitis media were more frequently diagnosed in children colonized with SPn. Children attending day care centres had significantly higher prevalence of SPn colonization (270 of 367, 44.4 % vs 338 of 533, 33.1 %, *p* = 0.001). Children with pneumonia, sinusitis and acute otitis media were more frequently treated with antimicrobials than children with other diagnoses.

**Conclusions:**

SPn nasopharyngeal colonization has a negative impact on the course of respiratory tract infection, likely because of SPn being the cause of the disease or a complicating factor. It is also associated with and may be responsible for higher frequencies of bronchitis, pneumonia, acute otitis media, sinusitis and the need of antimicrobial treatment.

## Background

*Streptococcus pneumoniae* (SPn) is one of the leading pathogens causing a variety of clinical manifestations especially among young children. It is responsible for around 11 % of deaths in children under the age of 5 years worldwide [1]. SPn colonizes the nasopharynx (NP) [2] and it may be asymptomatic or result in diseases such as upper respiratory tract infection (URTI), acute otitis media (AOM), sinusitis, pneumonia, sepsis and meningitis which are a considerable burden [1]. Most of these diseases often require antimicrobial treatment and SPn has shown increasing resistance to antimicrobials which is another public health concern [3–5].

Many recent studies emphasize the contribution of SPn to severe diseases such as pneumonia, bacteraemia, meningitis [6–12], and AOM [13–17], and the ability of pneumococcal conjugate vaccines to prevent them. Our study is somewhat novel in this regard that we examined the influence of SPn nasopharyngeal colonization on common respiratory tract infections (RTI), a cause of which may be SPn. Preschool children often present with RTI at their primary care physicians. Cough, fever, symptoms referable to throat, and earache are among the 20 leading principal reasons for outpatient visits [18]. The nasopharynx is the reservoir for SPn and the carriage of SPn in children with acute RTI has not been studied widely. This study was undertaken to evaluate the circulation of SPn serotypes among children under 6 years of age with acute RTI in Lithuania before the introduction of universal pneumococcal vaccination in the country in October 2014 [19]. This paper is an extension to the other results of our study which were published separately [20].

The possible associations of SPn nasopharyngeal colonization with the recovery time during the RTI, clinical signs and symptoms, and treatment with antimicrobials were analysed in our study. . Day care centres (DCC) and young siblings are known risk factors for SPn colonization [21, 22], and associations of DCC attendance and young siblings with SPn colonization were also evaluated.

As we did not test for other pathogens in the nasopharynges of our subjects, we could not determine the aetiology of the disease. We hypothesised that the RTI of children colonized with SPn (who are at risk of developing pneumococcal disease) is longer and more severe than RTI of non-colonized children. Nasopharyngeal colonization with SPn increases one’s risk of developing pneumococcal disease [23]. The most common cause of RTI is viral, which may facilitate the conversion of asymptomatic carriage of SPn to a pathogen and pneumococcal disease may be more severe than that caused by other pathogens.

## Methods

Children presenting with RTI at seven primary care centres in five major cities in Lithuania (Vilnius, Kaunas, Panevezys, Alytus, Klaipeda) and the Emergency Department of Children’s Hospital, Affiliate of Vilnius University Hospital Santariskiu Klinikos were included in our study. Vilnius Regional Biomedical Research Ethics Committee (Lithuania) approval was obtained (2011-11-08 No. 158200-11-418-118) and the parents or legal representatives were asked to sign an informed consent form before the child was enrolled in the study. The study was carried out from February 2012 to March 2013.

All subjects satisfied all of the following criteria at the study entry:

- Aged 0–71 months old at the day of sampling;
- Visited the primary health care provider (general practitioner or paediatrician) because of either upper or lower acute respiratory tract infection (fever of 37.2^0^ C or higher, and/or symptoms of respiratory tract infection: coryza, sore throat, cough, sneezing);
- Symptoms lasted no longer than 1 week from onset;

Signed by parents or legal representatives of the Patient Informed Consent and Written Agreement to participate in the study form. Subjects applying for any of the exclusion criteria listed below were not included in the study:

- Another known cause of fever is identified (e.g. confirmed infection other than RTI, such as urinary tract infection, etc.);
- History of vaccination with any pneumococcal vaccine (because of the vaccine’s effect on reducing colonization with vaccine serotypes [24]);
- Pre-treatment with antimicrobials for current RTI or treatment with antimicrobials within one month prior the enrolment, so as not to diminish the yield of nasopharyngeal culture samples.

General information, demographic data (date of birth, date of examination, siblings aged less than 6 years, DCC attendance, and prior use of antimicrobials) were collected and recorded to the Case report forms. The signs and symptoms assessed by the physician were fever, cough, coryza, lung auscultation findings, and inflammation of the pharynx and the palatine tonsils. Diagnoses were coded according to the International Classification of Diseases 10th edition Australian Modification (ICD-10-AM), which is used in all healthcare institutions in Lithuania as per requirement of the Ministry of Health.

Nasopharyngeal swabs were taken during the initial visit using culturette with Amies (Deltalab, Spain) transport medium and transported to a certified bacteriology laboratory of Children’s Hospital, Affiliate of Vilnius University Hospital Santariskiu Klinikos in Vilnius within 48 h from collection. Hare et al. compared several methods for NP samples transport and found Amies transport medium adequate for SPn detection [25]. Classic cultural methods (cultivation in 5 % CO_2_, colony morphology, optochin sensitivity) were used to isolate SPn from the swabs [26, 27]. We were unable to perform neither bile solubility, nor latex agglutination tests, however, we believe that the small number of potentially lost positive samples did not influence the accuracy of our results due to the large sample size of our study [28]. Serotypes were determined by means of latex agglutination reaction using Pneumotest-Latex kit (Statens Serum Institute, Copenhagen, Denmark).

Children were followed-up either via phone call or an additional visit during a 4 week period. The data obtained during the follow-up were final determination of diagnosis, duration of illness (recovery duration), treatment with antimicrobials, and number of days absent from DCC. Physicians were notified of the NP culture results after the follow-up.

Data were processed with the IBM SPSS version 18. Univariable Poisson regression with robust parameter estimates was used to analyse the associations of SPn colonization with sex, young siblings, clinical signs and symptoms, recovery duration (groups of 1–2, 3–4, and over 4 weeks; participants who failed to recover within 1–2 weeks were considered having longer recovery duration), treatment with antimicrobials during current disease, and particular diagnoses (URTI, bronchitis, pneumonia, sinusitis, AOM). Multivariable Poisson regression was used to assess the prevalence of SPn colonization in different age groups (0–23, 24–47, and 48–71 months), adjusted for DCC attendance. Cross tabulation with chi-squared test was used to assess the following: associations of sex with SPn colonization, longer recovery duration, and treatment with antimicrobials; associations of particular serotypes/serogroups with longer recovery duration, and treatment with antimicrobials (compared to non-colonized cases); associations of specific diagnoses (URTI, AOM, sinusitis, pneumonia, bronchitis) with treatment with antimicrobials. Student’s *T* test was used to compare the means of days absent from DCC of SPn colonized participants with non-colonized. Values of p less than 0.050 were considered statistically significant.

## Results

*Population*. A total of 900 children were included in our study. There were 492 (54.7 %) male and 408 (45.3 %) female subjects. The prevalence of SPn colonization was 40.8 % (*n* = 367), and did not differ significantly between female and male participants (168 of 408, 41.2 % and 199 of 492, 40.4 % respectively, *p* = 0.825). The specifics of results from participating facilities are presented in Table 1.

**Table 1:** Demographic specifics of the study population

	Site						Total
	ED^a^	Vilnius	Kaunas	Panevėžys	Alytus	Klaipėda	
Samples, n	264	173	159	223	18	63	900
Positive samples^b^, *n* (%)	121 (45.8)	86 (49.7)	52 (32.7)	82 (36.8)	2 (11.1)	24 (38.1)	367 (40.8)
Mean age, months (SD)	33.52 (16.45)	32.32 (16.33)	34.35 (18.84)	37.91 (17.57)	49.13 (15.37)	39.00 (18.57)	35.20 (17.45)
Males, *n* (%)	154 (58.3)	89 (51.5)	81 (50.9)	120 (53.8)	14 (77.8)	34 (54.0)	492 (54.7)
Females, *n* (%)	110 (41.7)	84 (48.5)	78 (49.1)	103 (46.2)	4 (22.2)	29 (46.0)	408 (45.3)
DCC attendance, *n* (%)	149 (56.7)	110 (64.7)	113 (71.1)	177 (79.4 %)	14 (87.5)	45 (73.8)	608 (67.6)
Antimicrobial use, *n* (%)	98 (39.2)	74 (43.3)	59 (37.3)	112 (50.2)	1 (12.5)	17 (28.8)	361 (40.1)

^a^ED–Emergency Department of Children’s Hospital, Affiliate of Vilnius University Hospital Santariskiu Klinikos. ^b^ Positive nasopharyngeal samples for *Streptococcus pneumoniae*

*Age*. We have selected three groups based on the age of the participants: 1) aged less than 24 months; 2) 24 through 47; and 3) 48 through 71 months old. Prevalence of SPn colonization was similar among all three groups when adjusted for DCC attendance (Table 2).

**Table 2:** Prevalence of SPn colonization in relation to various factors during respiratory tract infection

Characteristic		Colonized (*n* = 367)	Non-colonized (*n* = 533)	PR (95 % CI)	*P* value
Age (months) ^a^:	<24^c^	90	158		
	24–47	196	222	1.050 (0.813–1.356)	0.840
	48–71	75	137	0.736 (0.542–0.999)	0.049
Sex	Male	199	293	0.982 (0.939–1.150)	0.824
	Female^c^	168	240		
Siblings		145	189	1.114 (0.949–1.308)	0.187
DCC attendance^b^		270	338	1.423 (1.105–1.832)	0.006
Signs and symptoms:	Coryza	276	390	0.962 (0.753–1.230)	0.757
	Cough	240	331	1.133 (0.884–1.452)	0.323
	Fever	213	304	0.919 (0.740–1.143)	0.449
	Tonsils	191	260	1.074 (0.882–1.308)	0.478
	Auscultation	62	102	1.391 (0.881–2.197)	0.157
	Throat	295	416	1.087 (0.693–1.704)	0.716
Recovery:	1–2 weeks^c^	213	382		
	3–4 weeks	114	98	1.502 (1.274–1.771)	< 0.001
	> 4 weeks	8	12	1.117 (0.646–1.932)	0.691
Antimicrobial use		204	157	1.901 (1.618–2.234)	< 0.001
Diagnoses	URTI^c^	208	384		
	Bronchitis	62	51	1.562 (1.279–1.907)	< 0.001
	Pneumonia	14	10	1.660 (1.164–2.369)	0.005
	Sinusitis	23	13	1.818 (1.390–2.379)	< 0.001
	AOM	35	41	1.311 (1.004–1.712)	0.047
	Others	5	7	1.186 (0.602–2.337)	0.622
	Non-specified	20	27	1.211 (0.854–1.718)	0.283

PR (95 % CI)–prevalence ratio and 95 % confidence interval of *Streptococcus pneumoniae* nasopharyngeal colonization

Coryza–nasal secretion and/or nasal congestion

Fever–body temperature above 37.0 °C

Throat–signs of inflammation of the throat, including redness, oedema, and papules

Tonsils–signs of inflammation of the palatine tonsils, including redness, oedema, coating, and blisters

Auscultation–auscultation of the lungs, including signs of obstruction, rales, and crackles

^a^Adjusted for DCC attendance

^b^Adjusted for age

^c^Refference group for each comparison

*Siblings*. At least one sibling under the age of 6 years was present in the family in 334 (37.1 %) cases. The prevalence of SPn colonization in this group did not differ significantly compared to children who have no siblings (145 of 334, 43.4 % vs 219 of 562, 39.0 %, *p* = 0.190) (Table 2).

*Signs and symptoms*. We evaluated if there were any associations of fever, cough, coryza, lung auscultation findings, and inflammation of the pharynx and the palatine tonsils at the initial visit with SPn colonization. None of the signs and symptoms was predictive of SPn colonization (Table 2).

**Fig. 1:**
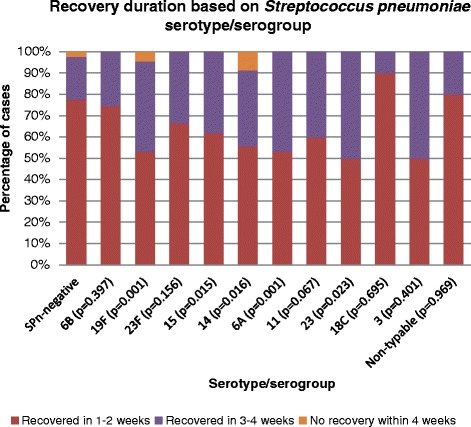
Recovery duration based on *Streptococcus pneumoniae* serotype/serogroup. Only serotypes with at least 10 cases are displayed. Recovery duration (1–2 vs 3–4 weeks vs longer than 4 weeks) for each serotype/serogroup compared with SPn-negative group (chi-squared test). 15–serogroup 15. 23–non-23F serotypes

*Recovery*. Data on recovery duration was available for 827 (91.8 %) cases. Three groups based on recovery duration were distinguished: 1) subjectrecovered within one to two weeks (shorter duration), 2) within 3 to 4 weeks (longer duration), and 3) no recovery within 4 weeks. Significantly more children colonized with SPn had longer recovery duration (of 3 to 4 weeks) compared to the children with negative cultures (114 of 335, 34.0 % vs 98 of 492, 19.9 % respectively, *p* < 0.001). There was a relatively small number of subjects who did not recover within 4 weeks and the proportions were similar in SPn positive (8of 335, 2.4 %) and negative groups (12 of 492, 2.4 %). Serotypes 19F, 14, 6A, and serogroups 15 and 23 (non-23F serotypes) were associated with longer recovery duration compared to non-colonized cases (Fig. 1).

*Diagnoses*. URTIs were diagnosed in 592 (65.8 %) cases (ICD-10-AM codes J00 and J02-J06). Bronchitis, pneumonia, sinusitis and AOM (ICD-10-AM codes J20, J18, J01, and H65-H66 respectively) were diagnosed in 249 (27.6 %) cases. There were 12 cases (1.3 %) with other specified diagnoses and 47 cases (5.2 %) had no diagnosis specified. Significantly more URTIs were diagnosed in the SPn-negative than in the SPn-positive group (384 of 533, 72.0 % vs 208 of 367, 56.7 % respectively, *p* < 0.001). The frequencies of diagnoses are presented in Table 3.

**Table 3:** Frequencies of diagnoses per *Streptococcus pneumoniae* serotype and use of antimicrobials per diagnosis

		Diagnosis, *n* (%)							Total, *n* (%)
		Unspecified	Other	AOM	URTI	Sinusitis	Pneumonia	Bronchitis	
Serotype	None	27 (5.1)	7 (1.3)	41 (7.7)	384 (72.0)	13 (2.4)	10 (1.9)	51 (9.6)	533 (59.2)
	Other^a^	1 (4.0)	0 (0.0)	3 (12.0)	13 (52.0)	4 (16.0)	1 (4.0)	3 (12.0)	25 (2.7)
	11	0 (0.0)	2 (11.8)	0 (0.0)	9 (52.9)	0 (0.0)	0 (0.0)	6 (35.3)	17 (1.9)
	14	2 (5.7)	0 (0.0)	3 (8.6)	15 (42.9)	2 (5.7)	5 (14.3)	8 (22.9)	35 (3.8)
	15^b^	2 (5.4)	0 (0.0)	3 (8.1)	25 (67.6)	1 (2.7)	2 (5.4)	4 (10.8)	37 (4.1)
	18C	2 (18.2)	0 (0.0)	2 (18.2)	5 (45.5)	1 (9.1)	0 (0.0)	1 (9.1)	11 (1.2)
	19A	1 (12.5)	1 (12.5)	0 (0.0)	4 (50.0)	1 (12.5)	0 (0.0)	1 (12.5)	8 (0.9)
	19F	4 (7.8)	0 (0.0)	9 (17.6)	25 (49.0)	3 (5.9)	1 (2.0)	9 (17.6)	51 (5.6)
	23^c^	0 (0.0)	0 (0.0)	0 (0.0)	5 (50.0)	3 (30.0)	0 (0.0)	2 (20.0)	10 (1.1)
	23F	1 (2.0)	0 (0.0)	1 (2.0)	35 (68.6)	5 (9.8)	2 (3.9)	7 (13.7)	51 (5.7)
	3	1 (9.1)	0 (0.0)	3 (27.3)	4 (36.4)	1 (9.1)	0 (0.0)	2 (18.2)	11 (1.2)
	6A	2 (5.9)	0 (0.0)	6 (17.6)	19 (55.9)	1 (2.9)	1 (2.9)	5 (14.7)	34 (3.8)
	6B	3 (5.2)	2 (3.4)	4 (6.9)	33 (56.9)	1 (1.7)	2 (3.4)	13 (22.4)	58 (6.4)
	Non-typable	1 (5.2)	0 (0.0)	1 (5.3)	16 (84.2)	0 (0.0)	0 (0.0)	1 (5.2)	19 (2.1)
Total, *n* (%)		47 (5.2)	12 (1.3)	76 (8.4)	592 (65.8)	36 (4.0)	24 (2.7)	113 (12.6)	900 (100.0)
Antimicrobial use, *n* (%)		14 (29.7)	3 (25.0)	55 (72.4)	173 (29.2)	32 (88.8)	23 (95.8)	61 (12.6)	361 (40.1)

*AOM* acute otitis media, *URTI* upper respiratory tract infection

^a^Combined cases of SPn serotypes 4, 7, 7F, 6C, 9, 9V, 10, 12, 17, 19, 22

^b^Serogroup 15

^c^Non-23F serotypes

*Serotypes*. The most frequent serotypes were 6B (15.8 %, *n* = 58), 19F (13.9 %, *n* = 51), 23F (13.9 %, *n* = 51), 15 (10.1 %, *n* = 37), 14 (9.5 %, *n* = 35), 6A (9.3 %, *n* = 34). Of the 367 SPn isolates 19 (5.2 %) were non-typable. The largest proportions of AOM were among the children colonized with serotypes 3, 6A, 18C, and 19F (Table 3). Non-23F serotypes were associated with sinusitis in 30.0 % (*n* = 3) of cases. Pneumonia was most frequently diagnosed in children colonized with serotype 14 (*n* = 5, 14.3 %).

*Day care centre attendance*. There were 608 children who were attending DCC. The prevalence of SPn colonization was significantly higher in children who were attending DCC than those who were not (270 of 608, 44.4 % vs 94 of 284, 33.1 %, *p* = 0.001). We also found that children with positive SPn cultures were longer absent from DCC due to the RTI. Females with positive SPn cultures were absent for 1.9 days longer (95 % CI: 0.042–3.764, *p* = 0.045); males–1.0 days longer (95 % CI: −0.937–3.052, *p* = 0.298); both sexes combined–1.5 days longer (95 % CI: 0.138–2.867, *p* = 0.031). Children with diagnoses other than URTI were on average for 4 days longer absent from DCC than children diagnosed with URTI (95 % CI: 2.562–5.448, *p* < 0.001).

*Antimicrobial use*. There were 212 children (23.6 %) who were treated with antimicrobials 1 to 6 months prior the NP sample. Prevalence of SPn colonization did not differ significantly between children who were treated with antimicrobials 1 to 6 months prior the NP sample and children who were not (98 of 212, 46.2 % vs 249 of 628, 39.6 %, *p* = 0.093). Data on treatment with antimicrobials during current illness was available for 869 patients (96.5 %). Among these patients 335 (37.2 %) had one course of antimicrobial treatment prescribed, and 26 (2.9 %) had two.

Significantly more subjects with SPn colonization were treated with antimicrobials during current episode of RTI compared to children with negative SPn cultures (204 of 355, 57.5 % vs 157 of 514, 30.5 %, *p* < 0.001) (Table 2).

**Fig. 2:**
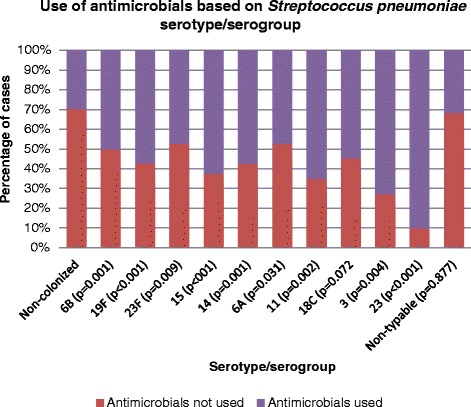
Use of antimicrobials based on *Streptococcus pneumoniae* serotype/serogroup. Only serotypes with at least 10 cases are displayed. Antimicrobial use (used vs not used) for each serotype/serogroup compared with SPn-negative group (chi-squared test). 15–serogroup 15. 23–non-23F serotypes

Antimicrobials were similarly prescribed to female and male participants (168 cases, 42.6 % and 188 cases, 40.4 % respectively, *p* = 0.512). Children with pneumonia, sinusitis and AOM were more frequently treated with antimicrobials than children with other diagnoses. The rates of antimicrobial prescription for specific diagnoses are presented in Table 3 and antimicrobial use per specific serotypes cases is shown in Fig. 2.

The prescribed antimicrobials were: amoxicillin (*n* = 122, 57.5 %), clarithromycin (*n* = 60, 28.3 %), phenoxymethylpenicillin (*n* = 48, 22.6 %), amoxicillin with clavulanate (*n* = 44, 20.7 %), cefuroxime (*n* = 34, 16.0 %), cefadroxil (*n* = 27, 12.7 %), azithromycin (*n* = 19, 8.9 %), ampicillin with sulbactam (*n* = 10, 4.7 %), trimethoprim with sulphamethoxazole (*n* = 6, 2.8 %), and erythromycin (*n* = 3, 1.4 %).

## Discussion

Respiratory tract infections are among the most common reasons for children to seek medical advice and treatment at the primary care physician. We studied the influence of nasopharyngeal colonization with SPn on the clinical course of RTIs. SPn nasopharyngeal colonization has important effects on the health of children and adults as it increases the risk of pneumococcal infection. While SPn is known to be the causative agent of pneumonia, AOM, sinusitis, various URTI and invasive pneumococcal diseases, there is little data on the influence of nasopharyngeal colonization with SPn on the course of RTI. DCCs are the main factor of pneumococcal transmission in a population as extensively shown by Hoti et al. [22]. DCC attendance predisposes children to SPn colonization which may spread among their families and then further within the adult population. Our results are in accordance to previous findings as we found DCC attendees having higher prevalence of SPn colonization [22, 29]. Interestingly, while the majority of sinusitis cases in our sample were among DCC attendees, it was not the case for pneumonia or AOM.

The median duration of SPn carriage is estimated to be 31 days in adults and 60.5 days in children [2]. SPn nasopharyngeal carriage may be asymptomatic in certain conditions (such as a viral infection), however, it may facilitate the conversion of SPn from commensal to a pathogen [2]. Consequently nasopharyngeal colonization with SPn may lead to a more severe disease. While we could not determine the aetiology of the RTI from the data we obtained in our study, we found that nasopharyngeal colonization with SPn is associated with longer recovery duration in children under 6 years old. The study conducted by Kristo et al. in 2006 evaluated the effects of nasal colonization with pathogenic bacteria (including SPn) on the course of acute respiratory infections in children. Although they used nasal middle metal specimens to identify bacteria and studied children aged at least 6 years, their findings show that SPn colonization during acute RTI was predictive of a longer course of illness [30]. The longer disease duration results in more days absent from DCC. Although we found children colonized with SPn during acute RTI being absent from DCC for longer periods than children with negative NP cultures, the difference was of small magnitude (average 1.5 days longer). The longer disease duration and longer absence from DCC are likely to increase caregiver’s absence from work which is a significant burden to the families and society.

We found presence of siblings in families of our subjects having no impact on the prevalence of SPn colonization. However, we did not take into consideration the DCC attendance status of older siblings, a factor found to be significant for SPn spread to younger siblings by Otsuka et al. [29].

Furthermore, we found SPn colonization to be associated with higher frequencies of pneumonia, sinusitis, and AOM. These findings may imply that nasopharyngeal colonization with SPn predisposes children to diseases such as sinusitis, pneumonia and AOM, possibly by means of co-interaction of various potential pathogens (both viral and bacterial) in the nasopharynx of the host [2]. Our finding of more frequent RTI complications with pneumonia in children with nasopharyngeal colonization with SPn are in accordance with previous findings [31].

In our study 40.1 % of children were treated with antimicrobials. Amoxicillin was the most commonly prescribed antimicrobial constituting 57 % of all prescriptions. In general, antimicrobials were appropriately used with majority of cases treated with antimicrobials being those of AOM, sinusitis and pneumonia, however, it should always be kept in mind that high antibiotic exposure is the most important cause for development of resistance to antimicrobials [32].

The majority of respiratory tract infections begin with non-specific symptoms such as fever, cough, and coryza. The additional signs evaluated by the physician (such as inflammation of the throat and palatine tonsils, and chest auscultation) are also non-specific and were not associated with SPn colonization in our study. Other researchers examined various possible features to aid in diagnosis of diseases caused by SPn: Chiappini et al. found no associations of signs and symptoms with aetiology of pneumonia [33], NP culture results were not found to be predictive of AOM by Casey et al. [14], and Shaikh et al. extensively studied the signs and symptoms in order to differentiate sinusitis and uncomplicated upper respiratory infections and found only green nasal discharge and sleep disturbances to be associated with radiological findings consistent with sinusitis [34]. These findings signify that the difficulties in distinguishing bacterial from viral causes of RTI remain high.

Vaccination with pneumococcal conjugate vaccines (PCV) reduces colonization rates of vaccine serotypes [35–38]. The protective effects of PCV were proven for invasive pneumococcal disease [12], pneumonia [11], and AOM [17, 39], while the PCV impact on sinusitis is not clearly defined [40]. The overall need of antimicrobial treatment is likely to be reduced with lower frequencies of these diseases caused by SPn. The higher rates of antimicrobial treatment for children with pneumonia, and AOM, and sinusitis and difficulties in clinically distinguishing children colonized with SPn point to the importance of pneumococcal vaccines as a measure to prevent diseases and sustain susceptibility of SPn to antimicrobials.

The major limitation of our study is that we did not test for other pathogens in the NP of our subjects, thus we could not determine the actual cause of the RTI. Nasopharyngeal colonization with SPn increases the risk of developing pneumococcal disease (either primary or as a complication of a previous RTI caused by another pathogen) and this is the most likely reason of our findings. An extended study with testing for other pathogens in the respiratory tract is necessary to confirm this. Another limitation is that we were unable to perform neither bile solubility nor latex-agglutination tests with negative NP samples and thus up to 3 % of our negative samples were potentially false-negative.

## Conclusions

SPn nasopharyngeal colonization has a negative impact on the course of respiratory tract infection, likely because of SPn being the cause of the disease or a complicating factor. It is also associated with and may be responsible for higher frequencies of bronchitis, pneumonia, acute otitis media, sinusitis and the need of antimicrobial treatment. Our results illustrate the negative impact of SPn colonisation on RTI (longer duration and absence from DCC) and indirectly–importance of pneumococcal vaccines as a measure to prevent SPn infection.

## Abbreviations

SPn: *Streptococcus pneumoniae*
NP: nasopharynx
URTI: upper respiratory tract infection
AOM: acute otitis media
RTI: respiratory tract infection
DCC: day care centre
ICD-10-AM: International Classification of Diseases 10th edition Australian Modification
PCV: pneumococcal conjugate vaccine

## References

[1] O’Brien KL, Wolfson LJ, Watt JP, Henkle E, Deloria-Knoll M, McCall N (2009). Burden of disease caused by *Streptococcus pneumoniae* in children younger than 5 years: global estimates. Lancet.

[2] Shak JR, Vidal JE, Klugman KP (2013). Influence of bacterial interactions on pneumococcal colonization of the nasopharynx. Trends Microbiol.

[3] Song JH (2013). Advances in pneumococcal antibiotic resistance. Expert Rev Respir Med.

[4] Varon E (2012). Epidemiology of *Streptococcus pneumoniae*. Med Mal Infect.

[5] Dagan R, Givon-Lavi N, Leibovitz E, Greenberg D, Porat N (2009). Introduction and proliferation of multidrug-resistant *Streptococcus pneumoniae* serotype 19A clones that cause acute otitis media in an unvaccinated population. J Infect Dis.

[6] Centers for Disease Control and Prevention (CDC) (2010). Invasive pneumococcal disease in young children before licensure of 13-valent pneumococcal conjugate vaccine–United States, 2007. MMWR Morb Mortal Wkly Rep.

[7] von Gottberg A, Cohen C, de Gouveia L, Meiring S, Quan V, Whitelaw A (2013). Epidemiology of invasive pneumococcal disease in the pre-conjugate vaccine era: South Africa, 2003-2008. Vaccine.

[8] Tardivo S, Poli A, Zerman T, D’Elia R, Chiamenti G, Torri E (2009). Invasive pneumococcal infections in infants up to three years of age: results of a longitudinal surveillance in North-East Italy. Ann Ig.

[9] Soley C, Arguedas A (2009). Understanding the link between pneumococcal serotypes and invasive disease. Vaccine.

[10] Guevara M, Barricarte A, Gil-Setas A, Garcia-Irure JJ, Beristain X, Torroba L (2009). Changing epidemiology of invasive pneumococcal disease following increased coverage with the heptavalent conjugate vaccine in Navarre, Spain. Clin Microbiol Infect.

[11] Loo JD, Conklin L, Fleming-Dutra KE, Deloria Knoll M, Park DE, Kirk J (2014). Systematic review of the effect of pneumococcal conjugate vaccine dosing schedules on prevention of pneumonia. Pediatr Infect Dis J.

[12] Conklin L, Loo JD, Kirk J, Fleming-Dutra KE, Deloria Knoll M, Park DE (2014). Systematic review of the effect of pneumococcal conjugate vaccine dosing schedules on vaccine-type invasive pneumococcal disease among young children. Pediatr Infect Dis J.

[13] De Wals P, Erickson L, Poirier B, Pépin J, Pichichero ME (2009). How to compare the efficacy of conjugate vaccines to prevent acute otitis media?. Vaccine.

[14] Casey JR, Kaur R, Friedel VC, Pichichero ME (2013). Acute otitis media otopathogens during 2008 to 2010 in Rochester, New York. Pediatr Infect Dis J.

[15] Pelton SI (2005). Otitis media: re-evaluation of diagnosis and treatment in the era of antimicrobial resistance, pneumococcal conjugate vaccine, and evolving morbidity. Pediatr Clin North Am.

[16] Caeymaex L, Varon E, Levy C, Bechet S, Derkx V, Desvignes V (2013). Characteristics and outcomes of acute otitis media in children carrying *Streptococcus pneumoniae* or *Haemophilus influenzae* in their nasopharynx as a single otopathogen after introduction of the heptavalent pneumococcal conjugate vaccine. Pediatr Infect Dis J.

[17] Zhao AS, Boyle S, Butrymowicz A, Engle RD, Roberts JM, Mouzakes J (2014). Impact of 13-valent pneumococcal conjugate vaccine on otitis media bacteriology. Int J Pediatr Otorhinolaryngol.

[18] National Hospital Ambulatory Medical Care Survey (2010). 2010 Emergency department summary tables. Centers for Disease Control and Prevention.

[19] Ministry of Health of Lithuania. Lietuvos Respublikos vaikų profilaktinių skiepijimų kalendorius. [Calendar of prophylatic immunisations of children, Republic of Lithuania 2014 [July 07]]. Vilnius: MoH. [Accessed on 28 Sept 2015]. Lithuanian. Available from: http://www3.lrs.lt/pls/inter3/dokpaieska.showdoc_p?p_id=466527.

[20] Usonis V, Stacevičienė I, Petraitienė S, Vaičiūnienė D, Alasevičius T, Kirslienė J (2015). *Streptococcus pneumoniae* nasopharyngeal colonisation in children aged under six years with acute respiratory tract infection in Lithuania, February 2012 to March 2013. Euro Surveill.

[21] Regev-Yochay G, Raz M, Dagan R, Porat N, Shainberg B, Pinco E (2004). Nasopharyngeal carriage of *Streptococcus pneumoniae* by adults and children in community and family settings. Clin Infect Dis.

[22] Hoti F, Erasto P, Leino T, Auranen K (2009). Outbreaks of *Streptococcus pneumoniae* carriage in day care cohorts in Finland–implications for elimination of transmission. BMC Infect Dis.

[23] Bogaert D, De Groot R, Hermans PW (2004). *Streptococcus pneumoniae* colonisation: the key to pneumococcal disease. Lancet Infect Dis.

[24] O’Brien KL, Millar EV, Zell ER, Bronsdon M, Weatherholtz R, Reid R (2007). Effect of pneumococcal conjugate vaccine on nasopharyngeal colonization among immunized and unimmunized children in a community-randomized trial. J Infect Dis.

[25] Hare KM, Stubbs E, Beissbarth J, Morris PS, Leach AJ (2010). Swab transport in Amies gel followed by frozen storage in skim milk tryptone glucose glycerol broth (STGGB) for studies of respiratory bacterial pathogens. J Microbiol Methods.

[26] World Health Organization (WHO) (2011). Laboratory methods for the diagnosis of meningitis caused by Neisseria meningitidis, *Streptococcus pneumoniae*, and *Haemophilus influenzae*.

[27] Satzke C, Turner P, Virolainen-Julkunen A, Adrian PV, Antonio M, Hare KM (2013). Standard method for detecting upper respiratory carriage of *Streptococcus pneumoniae*: updated recommendations from the World Health Organization Pneumococcal Carriage Working Group. Vaccine.

[28] Nunes S, Sa-Leao R, de Lencastre H (2008). Optochin resistance among *Streptococcus pneumoniae* strains colonizing healthy children in Portugal. J Clin Microbiol.

[29] Otsuka T, Chang B, Shirai T, Iwaya A, Wada A, Yamanaka N (2013). Individual risk factors associated with nasopharyngeal colonization with *Streptococcus pneumoniae* and Haemophilus influenzae: a Japanese birth cohort study. Pediatr Infect Dis J.

[30] Kristo A, Uhari M, Kontiokari T, Glumoff V, Kaijalainen T, Leinonen M (2006). Nasal middle meatal specimen bacteriology as a predictor of the course of acute respiratory infection in children. Pediatr Infect Dis J.

[31] Vu HT, Yoshida LM, Suzuki M, Nguyen HA, Nguyen CD, Nguyen AT (2011). Association between nasopharyngeal load of *Streptococcus pneumoniae*, viral coinfection, and radiologically confirmed pneumonia in Vietnamese children. Pediatr Infect Dis J.

[32] van Bijnen EM, den Heijer CD, Paget WJ, Stobberingh EE, Verheij RA, Bruggeman CA (2011). The appropriateness of prescribing antibiotics in the community in Europe: study design. BMC Infect Dis.

[33] Chiappini E, Venturini E, Galli L, Novelli V, de Martino M (2013). Diagnostic features of community-acquired pneumonia in children: what’s new?. Acta Paediatr Suppl.

[34] Shaikh N, Hoberman A, Kearney DH, Colborn DK, Kurs-Lasky M, Jeong JH (2013). Signs and symptoms that differentiate acute sinusitis from viral upper respiratory tract infection. Pediatr Infect Dis J.

[35] Ozdemir H, Ciftci E, Durmaz R, Guriz H, Aysev AD, Karbuz A (2013). Nasopharyngeal carriage of *Streptococcus pneumoniae* in healthy Turkish children after the addition of PCV7 to the national vaccine schedule. Eur J Pediatr.

[36] Sharma D, Baughman W, Holst A, Thomas S, Jackson D, da Gloria Carvalho M (2013). Pneumococcal carriage and invasive disease in children before introduction of the 13-valent conjugate vaccine: comparison with the era before 7-valent conjugate vaccine. Pediatr Infect Dis J.

[37] Lee EK, Jun JK, Choi UY, Kwon HJ, Kim KH, Kang JH (2013). Nasopharyngeal carriage rate and serotypes of and antimicrobial susceptibility in healthy Korean children younger than 5 years Old: focus on influence of pneumococcal conjugate vaccination. Infect Chemother.

[38] Fleming-Dutra KE, Conklin L, Loo JD, Knoll MD, Park DE, Kirk J (2014). Systematic review of the effect of pneumococcal conjugate vaccine dosing schedules on vaccine-type nasopharyngeal carriage. Pediatr Infect Dis J.

[39] Eskola J, Kilpi T, Palmu A, Jokinen J, Haapakoski J, Herva E (2001). Efficacy of a pneumococcal conjugate vaccine against acute otitis media. N Engl J Med.

[40] Shapiro DJ, Gonzales R, Cabana MD, Hersh AL (2011). National trends in visit rates and antibiotic prescribing for children with acute sinusitis. Pediatrics.

